# Chimpanzees (*Pan troglodytes*) recognize that their guesses could be wrong and can pass a two-cup disjunctive syllogism task

**DOI:** 10.1098/rsbl.2024.0051

**Published:** 2024-06-12

**Authors:** Benjamin Jones, Josep Call

**Affiliations:** ^1^ School of Psychology and Neuroscience, University of St Andrews, St Andrews KY16 9AJ, UK

**Keywords:** possibility, certainty, reasoning, chimpanzee, primate

## Abstract

When chimpanzees search for hidden food, do they realize that their guesses may not be correct? We applied a post-decision wagering paradigm to a simple two-cup search task, varying whether we gave participants visual access to the baiting and then asking after they had chosen one of the cups whether they would prefer a smaller but certain reward instead of their original choice (experiment 1). Results showed that chimpanzees were more likely to accept the smaller reward in occluded than visible conditions. Experiment 2 found the same effect when we blocked visual access but manipulated the number of hiding locations for the food piece, showing that the effect is not owing to representation type. Experiments 3 and 4 showed that when given information about the contents of the unchosen cup, chimpanzees were able to flexibly update their choice behaviour accordingly. These results suggest that language is not a pre-requisite to solving the disjunctive syllogism and provides a valuable contribution to the debate on logical reasoning in non-human animals.

## 1. Introduction

Non-human animals and young children spontaneously prefer alternatives with more favourable odds in object search or population-to-sample choice tasks, yet fail to appreciate the unique value of a certain outcome (*p* = 1), choosing it approximately equally to an alternative with *p* = 0.50 [1–8]. This has led Leahy & Carey [9] to argue that, owing to lacking the language of modal concepts, animals and preverbal infants lack a full model of possibility and instead use a minimal model reliant on making single simulations of reality, which they act on without considering alternative possibilities. In a certain versus uncertain choice, the minimal agent simulates the location of the uncertain item, adds it to their model of the world and then chooses indiscriminately between the certain and the simulated certain outcomes, because in the eyes of the minimal agent, both are known.

Object search tasks, in which multiple containers are present but only some are baited, are a valuable tool to investigate modal concepts because certain choices may constitute evidence of logical reasoning. Presented with two containers and a single hidden food item, chimpanzees acted to maintain access to both containers while they searched, but only if they had not observed the baiting [10], a behaviour that conflicts with the minimal model. However, in a repetition of the three-cup two-item task (three containers, one certainly baited) [2], the same group of chimpanzees failed to choose the certain cup above chance levels [5], preventing the authors from rejecting Leahy & Carey’s hypothesis. This inconsistency could indicate either that chimpanzees struggle with tracking two items across four possible locations, or that maintaining access to both search locations is a form of information seeking and does not require simulating either scenario until the point of searching.

Crucially, for a full model of possibility, one must understand that possibilities are mutually exclusive, meaning that alternatives can be ruled out by receiving additional information. In adult humans, this is achieved via the ‘disjunctive syllogism’: a *or* b, *not* a, *therefore* b. While substantial evidence suggests that non-human primates are able to solve disjunctive syllogism tasks [5,10–12], Engelmann *et al*. [5], who tested chimpanzees in a four-cup two-item paradigm, noted that their results resembled 3 year olds in the inclusive disjunction (A or B, not A therefore B), but 5 year olds in the exclusive disjunction (A or B, A therefore not B), the reverse of the pattern found in children. In children, adult-like choice behaviour is often treated as adult-like reasoning, however, in non-humans, there may be additional constraints that mask reasoning capacities.

We tested 12 zoo-housed chimpanzees in a novel repeated-choice paradigm that retrospectively probed participants’ certainty in their answers in the absence of new information. Then tested whether, when given information about the contents of the unchosen cup, they were able to reason about the contents of their chosen cup and flexibly adjust their behaviour. Importantly, our two-cup design decreases task demands compared with traditional paradigms. Finally, we modified the paradigm to rule out the use of stimulus enhancement as a simpler explanation which did not require reasoning via the disjunctive syllogism.

## 2. Experiment 1

**Figure 1.**
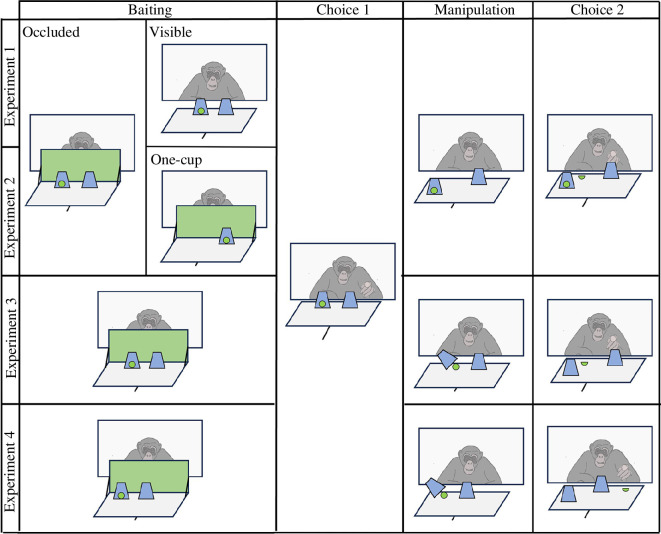
Schematic diagram of experiments 1–4. Experiments 3 and 4 show the remove empty condition (electronic supplementary material, video S1).

We tested nine chimpanzees (four female, mean age = 31.3 years, electronic supplementary material, table S1) at the Budongo Research Unit of Edinburgh Zoo. Subjects were socially housed, and testing took place opportunistically in a group setting in the research area. We applied a paradigm of post-decision wagering [13] to a basic two-cup task to test the level of confidence that subjects had in their choices. First, we baited with one whole grape one of two upturned cups either in view or behind an occluder, and offered the participant a choice, sliding back the unchosen cup and replacing it with a half grape before offering subjects the choice again (figure 1). If, as predicted by the minimal model, participants only considered one option then we would not see any difference in confidence between trials where they saw the baiting and trials where they did not. Subjects received 12 *visible* and 12 *occluded* trials. Each 12-trial block was arranged pseudo-randomly and contained six *visible* trials and six *occluded* trials, counterbalanced by the order in which the cups were visited. Intercoder reliability based on the first and second choices for 15% of trials was perfect (kappa = 1, *n* = 59).

### (a) Results and discussion

**Figure 2.**
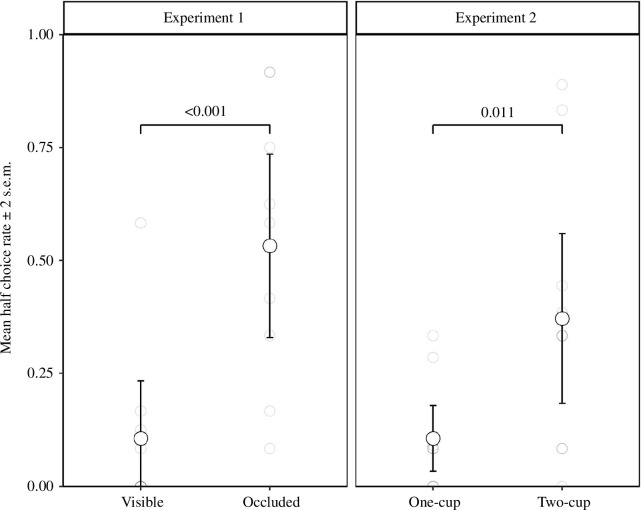
Group level differences in half choice rate based on conditions in experiments 1 and 2.

Figure 2 presents the per cent of trials in which subjects chose the half grape as a function of condition. Subjects chose the half grape significantly more often on visible trials than occluded trials (*t*‐test, *t*_8_ = 5.08, *p* < 0.001). Demonstrating that chimpanzees are not equating a guess with a certain outcome. Under the minimal model, a minimal agent will ‘use simulation to generate a single result and treat that result as reality’ [10, p. 67] therefore, the chimpanzees tested here are not conforming to the predictions of the minimal model. There was a large amount of interindividual variation in confidence (table 1), however, all individuals chose the half grape more frequently in the occluded than in the visible condition.

**Table 1.** Individual half choice rate by experiment and condition.

	experiment 1		experiment 2		experiment 3		experiment 4	
ID	visible	occluded	one-cup	two-cup	remove empty	remove baited	remove empty	remove baited
Edith	0	0.17	0.1	0.33	0.55	0.62	0	0.64
Eva	0.08	0.42	0.08	0.08	0	0.08	0	0.56
Frek	0	0.92	0	0.44	0.54	1	0.08	0.82
Kilimi	0	0.58	0.09	0.38	0.25	0.42	0.25	0.67
Louis	0.58	0.92	—	—	0.5	0.3	—	—
Lucy	0	0.08	0	0.08	0.08	0	0.33	0.58
Paul	0.12	0.62	0.33	0.33	—	—	0.23	0.82
Qafzeh	0.17	0.75	0.08	0.33	0.8	1	0.1	0.86
Velu	0	0.33	0	0	0	1	0	0.89
Liberius	—	—	0.29	0.89	—	—	—	—
Masindi	—	—	0.08	0.83	—	—	—	—
Sophie	—	—	—	—	—	—	0.20	0.57

However, Leahy & Carey [9] also argue that a minimal agent can behave in a way that resembles uncertainty monitoring without being aware of their uncertainty (also refer to [14] for a similar argument). They do so by learning to recognize perceptual features of a task or representation associated with a low frequency of success and opting out or seeking more information instead of running a simulation. Note, however, that post-decision wagering asks individuals to rate their decision retrospectively, so there is no option to opt out prior to the decision. So, once this simulation had been run and added to reality, in the absence of disconfirming evidence, there is no reason for it to be doubted.

However, it is possible that they may recognize the presence of the occluder, associating it with previous uncertainty-based tasks which they have engaged with, and decide before the point of choice that they would take the half grape without making a simulation. Moreover, the representation guiding the choice in the uncertain condition may be weaker than the representation formed in the certain condition. Recall that in the certain condition, the individuals saw the whole grape under one of the two cups but in the uncertain condition, they did not. This difference in representational strength may have caused a differential decrease in confidence, and the subsequent decision to opt out. We will test both of these explanations in experiment 2, by equating the presentations of the conditions to ensure that subjects respond based on the presence of conflicting representations, rather than the strength of the overall representation.

## 3. Experiment 2

Experiment 1 tested for a difference in confidence between a simulated representation and one obtained visually. In experiment 2, both conditions used a simulated representation, thus both have equivalent representational strengths. The two-cup condition was a replication of the occluded condition of experiment 1, with one of two cups baited with a whole grape behind an occluder, while the one-cup condition was also baited behind an occluder but there was only one cup available, so only one possible location for the grape. As in experiment 1, subjects received two sessions of 12 trials. Ten individuals participated in experiment 2 including two subjects who had not participated in experiment 1, Liberius, a 24 year old male and Masindi, a 3 year old female. Data were collected approximately 18 months after experiment 1. Intercoder reliability on 15% of trials was excellent (kappa = 0.982, *n* = 36).

### (a) Results and discussion

Figure 2 presents the percentage of trials in which subjects took the half grape in each condition, showing a significant difference by condition (*t*‐test, *t*_8_ = −3.18, *p* = 0.011), replicating the results of experiment 1. This demonstrates that chimpanzees differentiate between two generated representations based on the level of certainty that they produce, this would not be possible under even this more conservative reading of the minimal model.

Engelmann *et al*. [5] propose an alternative location-based argument for apes’ performance in three- and four-cup tasks, suggesting that subjects mark a broad location for each item, covering the range of cups it could be under, when a cup is revealed the subject can shrink or eliminate a location, but if both remain at the point of decision, they will pick indiscriminately between the two locations. This leads them to choose equally between the single cup, certainly containing a grape, and the pair of cups, in which each only has a 50% chance of containing a grape. In our task, the one-cup condition is theoretically equivalent to the certain cup in the three- and four-cup paradigms, while the two-cup condition is equivalent to the uncertain pair. If these two are valued equally, then we would see them chosen at the same rate against the half-piece, which we do not, instead, we see the subjects choosing in line with expected value.

Experiments 1 and 2 have provided evidence against the minimal model of possibility, experiments 3 and 4 will look at whether chimpanzees can reason via the disjunctive syllogism under this two-cup paradigm, which is considered to be evidence of modal reasoning.

## 4. Experiment 3

Eight individuals took part in experiment 3, which immediately followed experiment 1. The baiting procedure was the same as the occluded trials from experiment 1, but instead of dragging the unchosen cup backwards after the subject’s first choice, the experimenter lifted it and placed it at the back of the table. If the cup had been baited, they removed the grape and discarded it in a bucket on the ground; if it had been empty, they would look at the spot for 3 s before looking back to the centre. In both cases, the experimenter then placed the half grape where the non-chosen cup had been and slid the table to the subject. Experiment 3 did not include any certain trials. Provided that first choices were random (which they were, see below), the empty cup should be removed in approximately half of the trials and the baited cup in the other half. We refer to these as *remove empty* and *remove baited* trials, respectively. Subjects received two sessions of 12 trials. Intercoder reliability based on the first choice, removed cup contents and second choice for 15% of trials was excellent (kappa = 0.959, *n* = 29).

### (a) Results and discussion

**Figure 3.**
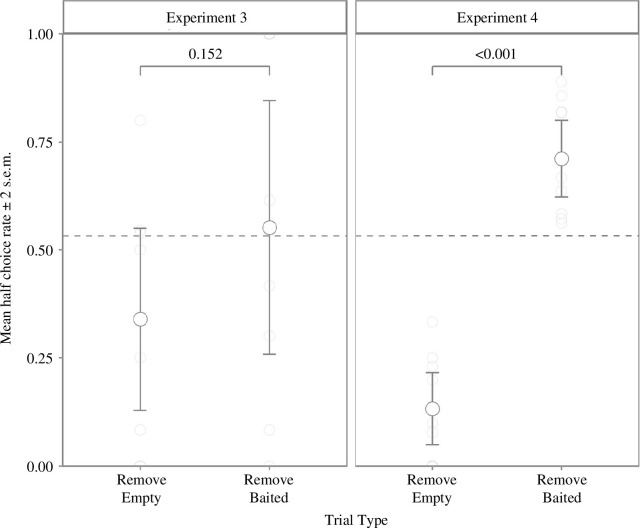
Mean rate of choosing the half grape by condition in the original and modified two-cup task.

From 192 trials, 95 were *remove baited* and 97 *remove empty*. Figure 3 presents the per cent of trials in which subjects took the half grape as a function of the contents of the unchosen cup, subjects chose the half grape more frequently on *remove baited* than *remove empty* trials, but not significantly so (*t*‐test, *t*_7_ = −1.61, *p* = 0.152).

To test for learning and main effects, we fitted a GLMM model (package: lme4) to predict the binary outcome of choosing the half grape (electronic supplementary material, table S3), using condition, session and the condition–session interaction as fixed effects, and individual ID as a random effect. The random effect of individual improved the fit over a GLMM including only the fixed effect structure (*χ* ^2^ = 59.63, d.f. = 1, *p* < 0.001) and the GLMM that included the fixed effects was an improvement over the null model containing only the random effect (*χ* ^2^ = 27.5, d.f. = 3, *p* < 0.001). The interaction term indicated diminished effect of condition in the second session, specifically through a reversion to chance in the *remove empty* condition (electronic supplementary material, figure S3). When we reanalyse only the first session, we see a significant difference between the conditions (*t*‐test, *t*_7_ = −2.99, *p* = 0.020).

Table 1 shows individual rates of taking the half grape across the four experiments. Fisher’s exact tests (electronic supplementary material, table S2) revealed a significant relation between the contents of the removed cup and choosing the half grape for two individuals in experiment 3, Frek (*p* = 0.016) and Velu (*p* < 0.001), who also both adapted their rate from experiment 1 in the predicted direction.

Notably, Velu answered correctly on all 24 trials of both conditions. The two conditions test the inclusive, A *or* B, *not* A *therefore* B, and the exclusive disjunction A *or* B, A *therefore* not B. Children can pass the inclusive disjunction at the age of 2.5 but cannot pass the exclusive disjunction until the age of 5 [7]. While individual differences are consistent with the primate literature [5,10–12] and success in one individual negates the suggestion that language is a necessary pre-requisite for solving the disjunctive syllogism, it may also reflect that that individual may have used a non-inferential strategy.

## 5. Experiment 4

In experiment 4, we modified the protocol of experiment 3 to counter the possibility of solving the task via stimulus enhancement. Instead of placing the half grape in the place of the removed cup, the experimenter placed it in a third position, either to the left or the right of the pair, counterbalanced between trials. Additionally, in the *remove empty* condition, the experimenter mimed the action of removing a grape to match their actions between conditions. Data were collected approximately 1 year after experiment 3. Nine individuals took part in this experiment including a 41 year old female (Sophie) who had not participated previously. Two individuals (Frek and Paul) received apple pieces instead of grapes, the large and small pieces were 1/16th and 1/32nd of an apple, respectively. Two individuals, Sophie and Velu only completed one block within the 12 available sessions, all others completed two full blocks of 12 trials. Intercoder reliability based on the first choice, removed cup contents and second choice for 15% of trials was excellent (kappa = 0.954, *n* = 32).

### (a) Results and discussion

From 192 trials, 103 were *remove baited* and 89 *remove empty.* Figure 2 shows the mean rates of choosing the half piece as a function of removed cup contents, the difference between conditions was significant (*t*‐test, *t*_8_ = −8.48, *p* < 0.001) as was the difference from chance (*remove empty*: *t*‐test, *t*_8_ = −8.18, *p* < 0.001; *remove baited*: *t*‐test, *t*_8_ = 4.77, *p* = 0.001). Moreover, Fisher’s exact tests showed that six of nine individuals correctly adapted their choice behaviour based on the contents of the unchosen cup (electronic supplementary material, table S2). These results suggest that, in a two-cup task, chimpanzees can solve both variants of the disjunctive syllogism.

Overall performance was significantly better in the modified version than the original (*t*‐test, *t*_6_ = −3.78, *p* = 0.009) (figure 2), however, rates of choosing the half-piece were not different in either the *remove empty* (*t*‐test, *t*_6_ = 1.55, *p* = 0.173) or the *remove baited* condition (*t*‐test, *t*_6_ = −1.01, *p* = 0.318). This suggests that the modified paradigm increased comprehension rather than biasing responses in one direction, potentially by increasing the salience of the half-choice as independent of the first choice. Fitting a GLMM as in experiment 3, we still find a main effect of condition on half choice rates, however, we do not find an interaction between session and condition, ruling out a learning effect (electronic supplementary material, figure S1 and table S4). The prolonged period between experiments 3 and 4, combined with a failure to find evidence of learning in either experiment would suggest that the results of experiment 4 are not a learned association. Notably, Velu remained close to ceiling in the modified task, suggesting that his performance in the original was inferential rather than associative and supporting the case for individual inference abilities existing on a spectrum in the great apes [15].

## 6. General discussion

It has been suggested that apes’ failure to appreciate a certain outcome is owing to an incapacity to discriminate *p* = 1 from *p* = 0.5 [2,16]. However, the data presented here challenge that conclusion. When choosing against a visible alternative of constant value, subjects altered their choice depending on how many cups were behind the barrier during the initial baiting. Presenting the options sequentially led to choices consistent with the expected value.

Using our simplified paradigm, we have demonstrated reasoning via both variants of the disjunctive syllogism, contrasting previous comparative literature [5,12], suggesting that procedural complexity may be the source of the divergence. Yet infants, who are capable of modelling small sets [17] and keeping the resulting representations separate from one another [18], also fail the four-cup task even after reducing task demands [8], so this cannot be the entire explanation. Nevertheless, reducing demands on executive function can positively impact apes’ performance on cognitive tasks [19,20] and in the three- and four-cup tasks subjects are simultaneously presented with two identical food pieces, one of which they must actively inhibit searching for. In contrast, in our two-stage task, subjects initially search for a single item, receive indirect feedback on that search and then choose to continue it or take the fractional piece. Future studies could apply our sequential procedure to the three- or four-cup paradigm to investigate whether this results in more rational choices.

While we have removed this constraint, a notable critique of two-cup one-item inference tasks is that they can be solved without understanding the concept of *therefore*, when A is shown empty the probability of B remains unchanged but becomes the only possible option. This experiment has shown that chimpanzees represent a dependency between the probabilities of A and B because, for multiple individuals, the frequency of a second behaviour (taking the half grape) was modulated by the contents of the revealed cup, a behaviour that corroborates previous findings [5,11,12]. Moreover, the lack of fixedness to one solution, which these chimpanzees have shown previously [21], demonstrates that this is an inferential rather than a learned response.

This means that apes not only consider that a food item might be located under cup A or cup B (abductive inference), but additionally, that the likelihood that the food item will be in cup B increases or decreases depending on whether they observe that cup A is empty or baited, respectively (inductive inference). However, this paradigm does not discern whether subjects are using deductive inference or inductive inference, and simply adjusting the *probability* that the remaining cup contains a grape and then choosing rationally between it and a half grape. The four-cup paradigm [6] also cannot rule out this explanation and future studies should explicitly aim to distinguish between inductive and deductive reasoning.

## 7. Conclusion

Taken together, these experiments demonstrate that chimpanzee choices are not governed by a singular simulation of reality and reflect the dependent relationship between mutually exclusive possibilities. The results of experiments 1 and 2 suggest chimpanzees are aware retroactively of their own uncertainty and have not been conditioned into opting out via punishments or time delays, and this cannot be explained by simple cues to uncertainty or differences in representational strength. Experiments 3 and 4 have demonstrated that, in a two-cup one-item version of the task, chimpanzees appear to reason via both variants of the disjunctive syllogism and that language is not necessary for this ability to emerge.

## Data Availability

Raw data, the code to reproduce the analysis and a description of the data can be found openly and permanently available at [22]. Data available as part of the supplementary material [23].
